# *PIEZO1* variant implications for biological understanding and human health

**DOI:** 10.1098/rsob.240345

**Published:** 2025-07-09

**Authors:** Chew W. Cheng, Sophie L. Earle, Oleksandr V. Povstyan, Chloe Randall, Katie A. Smith, Marjolaine Debant, Fraser L. Macrae, Daniel G. Beech, Anna McGrane, Fiona Bartoli, Eulashini Chuntharpursat-Bon, Richard M. Cubbon, Kathryn J. Griffin, Marc A. Bailey, Antreas C. Kalli, Lara C. Morley, Klaus K. Witte, David J. Beech

**Affiliations:** ^1^ Leeds Institute of Cardiovascular and Metabolic Medicine, School of Medicine, University of Leeds, Leeds LS2 9JT, UK; ^2^ Academic Department of Obstetrics and Gynaecology, School of Medicine, University of Leeds, Leeds LS2 9JT, UK; ^3^ Calderdale and Huddersfield NHS Foundation Trust, Huddersfield HD3 3EA, UK; ^4^ Department of Histopathology, Leeds Teaching Hospitals NHS Trust, Leeds, UK; ^5^ Leeds Vascular Institute, Leeds General Infirmary, Leeds, UK; ^6^ Department of Cardiology, Leeds Teaching Hospitals NHS Trust, Leeds, UK

**Keywords:** non-selective cation channel, calcium channel, calcium signalling, mechanical force, shear stress, endothelium

## Abstract

The large membrane protein PIEZO1 assembles as trimers to form exceptional mechanical force-sensing ion channels of eukaryotes. When these channels are activated by force, cell membrane permeability to calcium ions and other ions increases rapidly, coupling force to cell function through ionic control. In humans and other species, PIEZO1 is both widely expressed and functional across major systems that include the cardiovascular, haematological and musculoskeletal systems, thereby serving diverse needs. In this narrative review of the scientific literature, we address what has been learned about PIEZO1 from associations of its gene variation with human characteristics. A particular physiological importance of PIEZO1 is emerging in lymphatics and thus in the control of tissue fluid homeostasis with relevance to the disease conditions of non-immune fetal hydrops and generalized lymphatic dysplasia. Other vascular relevance is seen in lower limb venous varicosities. PIEZO1 may be non-essential in red blood cells but the amplification of its function by gene variation quite selectively alters these cells, leading to haemolytic anaemia and other related disturbances that may be only mildly adverse and confer survival advantage. We speculate on what else might be learned in humans, guided by knowledge from PIEZO1 studies in mice, and describe how knowledge accumulated to date highlights new opportunities for PIEZO1 understanding and pathways to patient benefit.

**Figure:**
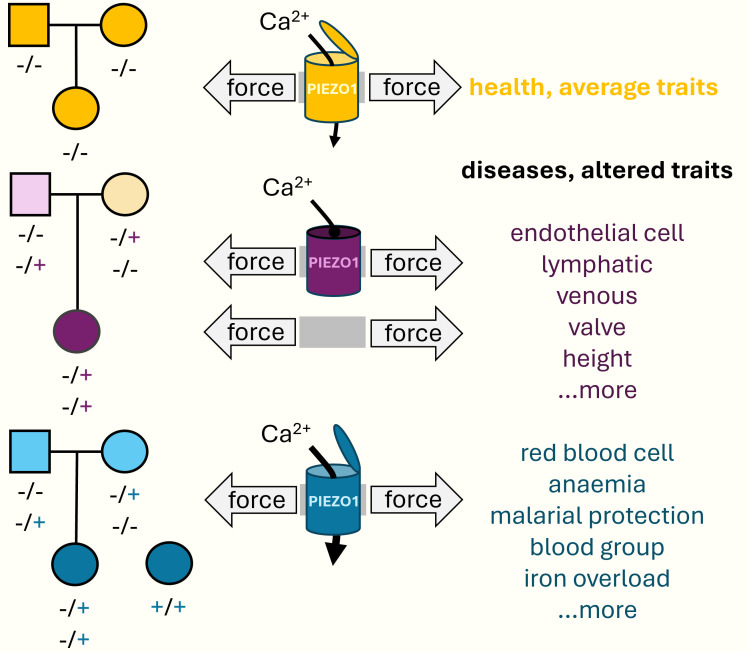

## 1. Introduction

**Figure 1.**
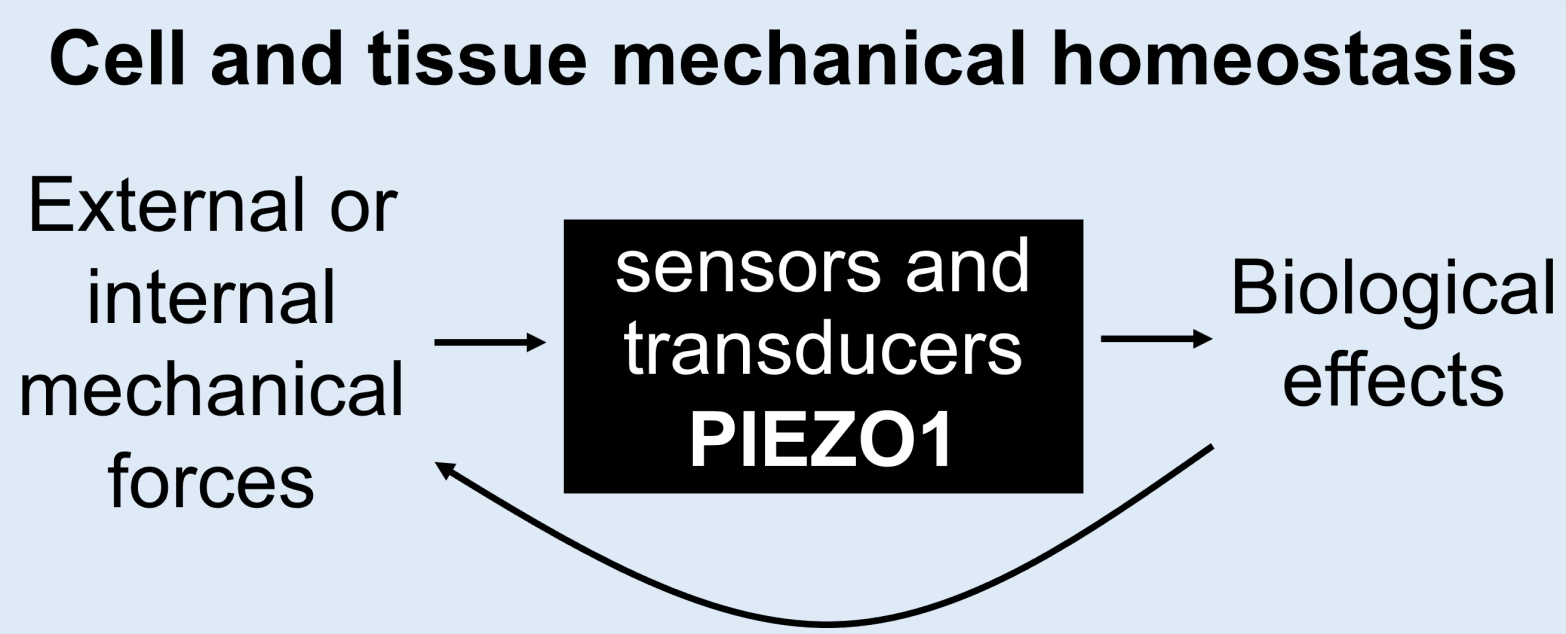
Illustration of the concept of mechanical homeostasis concept. The central box indicates the requirement for mechanical force sensors to detect force, and transducers to convert the amplitude of the detected force into a cellular effect. The molecular details of the sensors and transducers are not fully determined but PIEZO1 channels are both a crucial force sensor and transducer in many cell and tissue types.

Mechanobiology is the ability of biological molecules, cells and tissues to detect and respond to mechanical forces, thereby ensuring that the mechanical states of these structures match the external and internal demands of organisms (figure 1). Mechanobiology has been described as ‘the elephant in the room … overlooked’ [1], but there is now increasing recognition of its importance across animal biology including human health and disease [2–7]. Moreover, although all biological molecules are potentially mechanically sensitive depending on the intensity and type of stimulus, it has become increasingly clear that specific proteins evolved as dedicated force sensors of physiology, conferring high-fidelity force-dependent responsiveness [3,7]. A key protein of this type is PIEZO1 [8–10]. It has remarkable force sensing and transduction capabilities, serves force sensing needs in many cell and tissue types, and may be a ubiquitous force sensor across eukaryotes [8,10–12].

### 1.1. PIEZO1 properties

**Figure 2.**
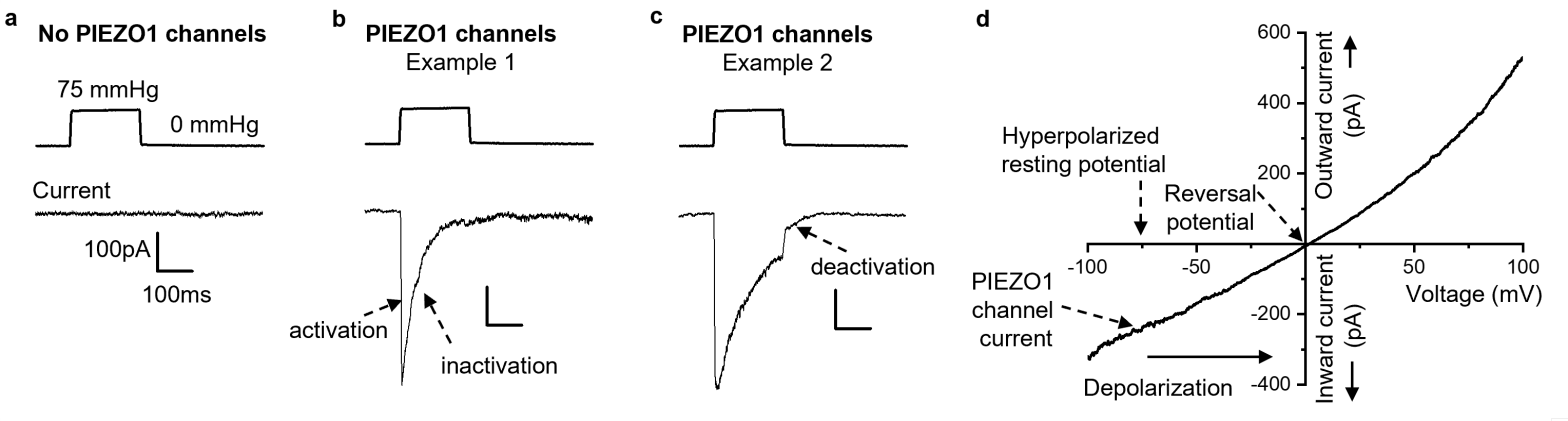
PIEZO1 channel electrophysiology. Patch-clamp data obtained from HEK 293 cells that were not transfected (a) or which were transfected (b–d) with plasmid containing cDNA encoding human PIEZO1 (hPIEZO1) channels. (a–c) Outside-out patch data at a constant transmembrane voltage of −80 mV (millivolt). (a–c) Example pressure steps applied to the patch are shown in the upper traces with associated current traces below for ionic currents across the membrane. All pressure steps were from a background of zero pressure (0 mmHg) to +75 mmHg (positive pressure on the patch pipette, leading to stretch of the membrane). The 100 pA (picoampere) and 100 ms (millisecond) calibrations apply to all current traces. (a) Without PIEZO1 transfection, no current was seen in response to the pressure step. (b,c) With PIEZO1 transfection, inward current (downward deflection) was seen in response to the pressure step. Two examples of the variation in the properties of the PIEZO1 channel currents are shown in (b) and (c). In example 1 (b), there was very fast activation and then slightly slower but still very fast inactivation that was complete within the time course of the pressure step (i.e. no PIEZO1 current remained). In example 2 (c), there was also fast activation but then slower inactivation that was incomplete (i.e. about 25% of the PIEZO1 current remained at the end of the pressure step). After the pressure step ended, deactivation of the remaining current occurred (c). (d) Whole-cell voltage-clamp data for current evoked by the PIEZO1 agonist Yoda1 and obtained in response to a ramp change in voltage from −100 mV to +100 mV. The current progressively changed from inward polarity at −100 mV to outward polarity at +100 mV with the cross-over point from inward to outward current referred to as the reversal potential. In physiology (without voltage-clamp), the resting membrane potential is expected to be in the region of −30 and −80 mV. Therefore, inward current through PIEZO1 channels (i.e. influx of cations, which are positively charged) causes depolarization towards the reversal potential.

PIEZO1 was first defined as a force sensor in patch-clamp recordings from nerve cancer cells cultured in an incubator [9]. The studies were possible because PIEZO1 forms calcium ion (Ca^2+^)-permeable non-selective cationic channels in the outer cell membrane [9]. The channels open in response to the application of experimentally applied forces such as increased pressure in the patch-clamp recording pipette [9,13]. As the channels open, they allow the flow of millions of ions across the cell membrane, creating a measurable ionic current (figure 2a–d). Although artificial methods of this type can activate the channels in experiments, the channels are also sensitive to physiological forces that arise, for example, from fluid flow [14] and tissue stiffness [15].

PIEZO1 channels change in their properties depending on their context but they usually activate quickly (in milliseconds or less) when there is a rapidly applied force (figure 2b,c). Channel activation is seen through a net flux of cations into cells at negative membrane potentials (figure 2d), which is the usual electrical polarity of cells. In the absence of experimentally applied voltage control, such ion influxes cause depolarization, a lessening of the negative membrane potential (figure 2d) that can increase or decrease cell function depending on the cell type. A cation that enters cells via PIEZO1 channels is Ca^2+^, which is a pivotal currency of intracellular control [16]. Its cytosolic elevation can trigger many cell activities such as contraction, secretion and migration [17]. The fluxes of two other physiological cations, the sodium ion (Na^+^) in and the potassium ion (K^+^) out, can also have specific impact. Therefore, PIEZO1 has the potential to alter cell function through several ionic mechanisms that modulate downstream processes, thereby generating effects.

Importantly, once the channels are activated, they can desensitize rapidly when there is sustained mechanical force (figure 2b). This property of inactivation is regulated, for example by lipid and protein factors [18–20], and so inactivation may be absent or occur slowly (figure 2c) [18]. Regulated inactivation seems to be a key way in which PIEZO1 channels achieve different functions in diverse cell types and contexts [20]. Once the mechanical stimulus is removed, the channels switch off through another process that is referred to as deactivation (figure 2c). Inactivation and deactivation are both a type of off switch but inactivation differs in its tendency to cause refractoriness to the subsequent opening of the channel when another stimulus arrives. Inactivation thus confers a memory of prior events.

**Figure 3.**
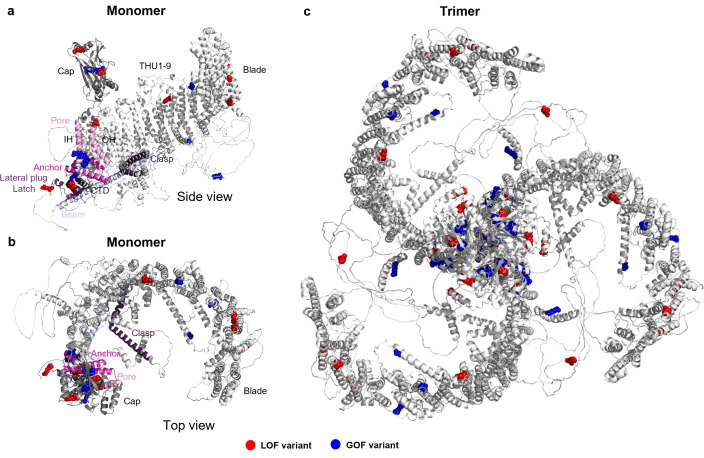
PIEZO1 channel structure: the AlphaFold (https://alphafold.ebi.ac.uk) model of human PIEZO1 AF-Q92508-F1-v4. (a,b) The monomer in side-view (a) and top (helicopter) view (b). The N-terminus is to the right and the C-terminus to the left. (c) The trimer (the complete PIEZO1 channel) assembled by aligning the single chain (monomer) of the AlphaFold model to the coordinates of all three chains of the full-length mouse PIEZO1 model in Pymol (www.pymol.org). The N-termini are at the outer tips of the blades and the C-termini are central. The trimer in (c) is artificially enlarged relative to the size of the monomer in (a,b) for visual presentation. (a–c) Highlighted in red or blue are variants that have been shown by patch-clamp to affect channel function: Red, loss of function (LOF); blue, gain of function (GOF). In the trimer, each variant is repeated three times, thus assuming homozygosity. Details of these variants can be found in the main text and table 1. (a,b) Indicated structural features of the channel: the cap; the pore-lining inner and outer helices (IH and OH) in light pink and the anchor in darker pink; the beam in light grey and the lateral plug and latch in darker grey; and the clasp and the CTD in dark grey. The blade comprises the 9 THUs, THU1−9.

The PIEZO1 channel is formed by three PIEZO1 proteins, each comprising about 2500 amino acid residues (2521 in human PIEZO1) arranged in chains that loop in and out of the membrane 38 times, leading to 38 membrane-spanning segments per monomer (figure 3a,b) and 114 for the whole channel trimer (figure 3c) [8,21,22]. The channel assembly embeds in and indents the membrane, forming a basket-like (dome) structure when in its closed ion-impermeable configuration [22,23]. Long tentacle-like blade structures span out from the core of each monomer (figure 3c). Each blade comprises nine transmembrane (TM) helical units (THU1−9), so there are 27 THUs per channel. Between THU7 and THU8 of each monomer is a linker on the intracellular side that includes a beam helix extending towards the pore (figure 3a). The beam is followed by a lateral plug and latch before an unstructured loop region and the clasp sitting below THU8 (figure 3a). After the last THU (THU9) is the anchor and then the outer helix formed by TM37, which lines the central ion pore with the inner helix of TM38. These two pore-lining helices are intervened by the C-terminal extracellular domain (CED), which sits above the central core, forming a cap that has foot-like structures touching nearby blade regions [22] (figure 3a). The pore is lastly cuffed on the intracellular side by the C-terminal domain (CTD) (figure 3a). Lateral tension in the membrane causes the dome to flatten and the blades to spread out radially. With flattening and expansion comes opening in the core of the channel and ion permeation, and thus a signal for the cell to act [8,23]. A more in-depth description of the channel structure can be found elsewhere [8,23], although much remains to be understood.

**Table 1.** Phenotypes associated with PIEZO1 protein variants. Listed in the Table are *PIEZO1* variants that affect the amino acid sequence of the PIEZO1 protein. The data are from peer-reviewed or preprint papers in the public domain. Additional variants may be found in databases as indicated in the main text. A few variants listed previously [8] could not be confirmed in our search of the peer-review literature, so they are not included. Amino acid residue numbers are for human PIEZO1 with the equivalent for mouse PIEZO1 in curved brackets, determined using a Clustal Omega alignment that compared UniProt (www.uniprot.org) mouse (E2JF22) and human (Q92508) PIEZO1 amino acid sequences. Structural regions of the human PIEZO1 channel are [8] (with amino acid numbers in square brackets): Blade Transmembrane Helical Units (THUs) 1−7 [1−142; 143−334; 345−541; 542−700; 701−948; 949−1116; 1117−1304]; Beam [1305−1367]; Central plug [1368−1401]; Lateral plug [1402−1411]; Latch [1412−1425]; Latch-to-Clasp [1426−1514]; Clasp [1515−1570]; Clasp-to-THU8 [1571−1656]; Blade THUs 8−9 [1657−1802; 1803−2098]; Anchor [2099−2165]; Outer helix [2166−2193]; Cap (C-terminal extracellular domain, CED) [2194−2439]; Inner helix [2440−2473]; C-terminal domain (CTD) [2474−2521]. Each PIEZO1 channel is a trimer of 3 PIEZO1 proteins, so the total number of amino acids per human PIEZO1 channel is 7563 and each variant repeats three times per channel in homozygosity. Amino acid numbers for structural regions of mouse PIEZO1 channel are specified elsewhere [8]. ‘None specified’ indicates where, to the best of our knowledge, no peer-reviewed data are available. The 'references' column indicates the underlying numbered reference containing the supporting evidence. Where laboratory data are available for the effect of the variant on channel function, the effect is indicated in blue for GOF and red for LOF or LOP. Abbreviations: hom. (homozygous); het. (heterozygous); c-het. (compound heterozygous); del. (deletion).

PIEZO1 amino acid number from N-terminus human (mouse)	amino acid or other change	PIEZO1 structural region		laboratory data for effect on PIEZO1	primary disease	antenatal and other clinical observations, interventions and outcomes	**references**
31 (31)	S to A frameshift (with R49 premature termination)	blade THU1		none specified	lymphatic malformation; may be classified as generalized lymphatic dysplasia (GLD)	non-immune fetal hydrops (NIFH) subcutaneous oedema in head and trunk hydrothorax termination of pregnancy	[31]
41 (41)	L deletion (with T1638 premature termination)	blade THU1		none specified	GLD	NIFH	[32]
49 (49)	R premature termination (with S31A)	blade THU1		none specified	NIFH lymphatic malformation (GLD?)	subcutaneous oedema in head and trunk hydrothorax termination of pregnancy	[31]
62−65 (62-65)	AHGK deletion	blade THU1		none specified	dehydrated hereditary stomatocytosis (DHS)	none specified	[33]
83 (83)	I to T	blade THU1		none specified	COVID-19	none specified	[34]
88 (88)	D to E	blade THU1		none specified	DHS with or without perinatal oedema	chylothorax serial thoracentesis maternal propranolol Iatrogenic abortion	[35]
103 (103)	R premature termination (c-het.)	blade THU1		none specified	NIFH GLD	none specified	[36]
152 (152)	P to L	blade THU2		none specified	COVID-19	none specified	[34]
189 (196)	R premature termination	blade THU2		none specified	lymphatic malformation (GLD?)	scalp, total body, skin and nuchal (neck) fold oedema bilateral pleural effusion ascites pericardial effusion Cardiomegaly, agenesis of the ductus venosus, placentomegaly, enlarged tongue, anemia percutaneous umbilical blood sampling with transfusion Intrauterine foetal demise	[35]
217 (224)	S to L	blade THU2		decreased activation protein degradation	bicuspid aortic valve disease	none specified	[37–39]
250 (257)	V to A (or I [40])	blade THU2		none specified	varicose vein (VV) COVID-19 alcoholic liver disease	none specified	[34,40,41]
251 (258)	A to T	blade THU2		none specified	severe congenital neutropenia	variant predicted to be benign	[42]
253 (260)	G to R (with S2195L)	blade THU2		decreased opening (mouse PIEZO1)	prune belly syndrome	abdominal wall laxity flared ribs dimpled knees dilated bladder bilateral hydroureter surgical urinary bladder drainage and other procedures bilateral hydronephrosis supraventricular tachycardia	[43]
315 (321)	S to I	blade THU2		none specified	inherited bone marrow failure	none specified	[44]
322 (329)	L to P (c-het. with E997 premature termination)	blade THU2		decreased mechanical sensitivity	NIFH	none specified	[45]
338 (345)	S to Y	blade THU3		none specified	DHS	none specified	[46]
376 (383)	P to A (het.)	blade THU3		none specified	congenital haemolytic anaemia	none specified	[47]
422 (429)	Q premature termination	blade THU3		none specified	NIFH	none specified	[48]
427−428 (434-435)	A-L insertion	blade THU3		none specified	DHS	none specified	[49]
457 (464)	R to C	blade THU3		none specified	DHS	none specified	[50]
499 (505)	V to I (with R2303H)	blade THU3		none specified	DHS lymphatic malformation (GLD?)	oedema	[51,52]
513 (519)	C to S frameshift (with S1540A)	blade THU3		none specified	NIFH	nuchal translucency	listed in [51,53]
531 (537)	R to C	blade THU3		decreased opening (mouse PIEZO1)	osteoarthritis	non-syndromic familial osteoarthritis free from acute or traumatic joint injury. erosive hand osteoarthritis and interphalangeal joint osteoarthritis	[54]
537 (543)	K to N	blade THU3		none specified	inherited bone marrow failure	none specified	[44]
598 (604)	V to M [55] or L [46]	blade THU4		increased opening [55] no change [56]	DHS GLD	NIFH termination of pregnancy [57] double superior vena cava [57]	[46,49,55–60]
599 (605)	V to G or L	blade THU4		none specified	DHS NIFH	termination of pregnancy pleural effusions, ascites with or without pseudohyperkalemia with or without perinatal oedema	[51,61,62]
605 (611)	M to V or I	blade THU4		none specified	DHS	none specified	[46,63]
655 (661)	Y premature termination (c-het. ?)	blade THU4		none specified	NIFH	pleural effusion and chylothorax, skin oedema medical termination of pregnancy placental hypertrophy	[51,64]
669 (675)	D to Y or H	blade THU4		none specified	DHS	potential association with cardiomyopathy [65]	[33,46]
679 (685)	E premature termination (hom.)	blade THU4		none specified	NIFH	none specified	[66]
681 (687)	F to S	blade THU4		no change	DHS	none specified	[46,56]
702 (708)	H to Y	blade THU5		none specified	adenomatous polyposis	none specified	[67]
718 (724)	G to S	blade THU5		none specified	DHS	none specified	[46,68]
722 (728)	P to L	blade THU5		none specified	brain arteriovenous malformations	none specified	[69]
745 (746)	Q deletion	blade THU5		none specified	DHS	none specified	[33]
755 (749)	E premature termination (with Q2228 premature termination)	blade THU5		decreased expression	GLD NIFH	pleural effusions polyhydramnios died *in utero* at 34 weeks mild generalized oedema with pleural effusions, chylothorax, periorbital oedema cupped simple ears, epicanthic folds, micrognathia, atrial septal defect, amyoplasia of the diaphragm, gastro-oesophageal reflux, short stature, pectus excavatum, genital oedema, splenomegaly	[70]
755−756 (749-750)	EE deletion	blade THU5		none specified	brain arteriovenous malformations	none specified	[69]
756 (750)	E deletion	blade THU5		increased activity (slowed inactivation)	malaria resistance in some populations [71,72] RBC dehydration increased transferritin saturation [25] increased ferritin [25] tendon stiffness and jumping ability in some populations	not associated with malarial resistance in some populations [73] not associated with sickle disease [74] not associated with glaucoma, blood pressure or body mass index [75].	[25,71–73,75–78]
782 (777)	G to S (with variants at 808 or 808 and 1764)	blade THU5		increased activity [56]	DHS with or without perinatal oedema	cystic hygroma liveborn dysmorphic features redundant nuchal skin fifth toe clinodactyly bilateral ventriculomegaly	[35,46,56,60,68]
808 (803)	R to Q or G (may be with 782 and 1764 variants)	blade THU5		no change (R808Q) [56]	DHS GLD	cystic hygroma with or without perinatal oedema liveborn dysmorphic features redundant nuchal skin fifth toe clinodactyly bilateral ventriculomegaly	[35,46,60,68,79]
829 (824)	E to V (with or without I2270T)	blade THU5		decreased activity and expression	GLD	bilateral hydrothoraces (het.) NIFH (het.) polyhydramnios (het.) oedema of lower limbs (E829V only) intermittent oedema of face, scrotal oedema (het.) pleural effusions/chylothoraces (het.) gastro-oesophageal reflux requiring fundoplication and gastrostomy (het.) Asperger syndrome, metopic, craniosynostosis, obstructive sleep apnoea bilateral periorbital and conjunctival vascular changes with small punctate haemorrhages (het.)	[80]
860 (855)	V to M	blade THU5		none specified	congenital hemolytic anaemia	none specified	[47]
868 (863)	C premature termination	blade THU5		none specified	NIFH	ascites, pleural effusion, skin oedema, hydramnios, polyhydramnios lymphedema, lymphangiectasia signs of maternal vascular malperfusion death at 1 month old (hom.)	[64]
870 (865)	M to I (c-het.)	blade THU5		none specified	NIFH DHS primary immune deficiency disease (het.)	suspected posterior urethral valve hepatic dysfunction	[32,51,59,81]
908 (903)	R to P	blade THU5		none specified	inherited bone marrow failure	none specified	[44]
915 (910)	N to S (c-het. with G6PD variant)	blade THU5		none specified	severe haemolytic anemia (DHS?)	none specified	[82]
939 (934)	L to M (with R2456C and F2458L)	blade THU5		decreased mechanical sensitivity [38]	NIFH DHS	pleural effusions polyhydramnios generalized oedema at birth, chylothorax/chylothoraces, webbed neck, periorbital oedema, prune belly gastro-oesophageal reflux, hypothyroid at birth, occasional stomatocytes	[46,70]
953 (948)	R to H (with exon 1−50 deletion)	blade THU6		none specified	lymphatic dysplasia (GLD?)	hydroceles, VVs, facial asymmetry, dysplastic ears, thoracolumbar scoliosis, mild hearing loss, mild intellectual disability, multiple fracture history, short stature, thoracolumbar scoliosis, left-sided facial bone hypoplasia, left ossicular malformation, prognathism	[83]
966 (961)	V to M	blade THU6		none specified	DHS	none specified	[84]
972 (966)	R to H	blade THU6		none specified	DHS	none specified	[62]
975 (970)	L to V	blade THU6		none specified	DHS With or without perinatal oedema	bilateral chylothorax ascites pleural effusion, respiratory distress, right lower quadrant venous lymphatic malformation, serial thoracentesis, chest tube placement, ventilation, propranolol, diuresis, liveborn	[35]
991 (986)	F to L	blade THU6		none specified	cerebral cavernous malformation	none specified	[85]
997 (992)	E premature termination (c-het. with L322P)	blade THU6		none specified	NIFH	none specified	[45]
1007 (1002)	I to M	blade THU6		none specified	adenomatous polyposis	none specified	[67]
1010 (1005)	R to H (with R1671W)	blade THU6		none specified	GLD	chylothorax bilateral lower limb oedema	[86]
1011 (1006)	M to I	blade THU6		none specified	inherited bone marrow failure	none specified	[44]
1069 (1064)	W premature termination (with K2070Q)	blade THU6		none specified	GLD NIFH	stillbirth facial and corporal oedema macroencephaly hypoplastic lungs failure of lymphatic luminal canalization hydroscopic chorionic villi fetal vascular malperfusion	[87,88]
1070 (1065)	R to C	blade THU6		none specified	lymphatic malformation (NIFH?)	increased nuchal fold bilateral pleural effusion total body and skin oedema ascites pericardial effusion polyhydramnios macrocephaly, anasarca, bilateral pleural effusion, hypospadias, and respiratory distress neonatal mechanical ventilation and bilateral chest tube placement neonatal death	[35]
1073 (1070)	R to W	blade THU6		none specified	erythrocytosis	none specified	[89]
1111 (1106)	Q premature termination (het.)	blade THU6		none specified	Er antigen	none specified	[90]
1114 (1109)	Q to E (with A2395V)	blade THU6		none specified	GLD	NIFH	listed in [51,53]
1117 (1112)	S to L (with A2020V)	blade THU7		none specified	DHS	none specified	[68]
1153 (1148)	S premature termination (with G2029R)	blade THU7		none specified	lymphatic dysplasia (NIFH?)	hydrops bilateral chylothorax swelling of legs and scrotum lymphedema of legs, torso and face pleural effusions intensive care including extracorporeal membrane oxygenation (one sibling)	[91]
1201 (1196)	T to M	blade THU7		none specified	neuro-developmental disorder	variant predicted to be non-pathogenic	[92]
1223 (1218)	V to I	blade THU7		none specified	DHS myelodysplastic syndrome	iron overload, splenomegaly, haemolysis	[93,94]
1247 (1241)	F to C	blade THU7		none specified	liver biliary pancreas abnormality	none specified	[40]
1255 (1250)	S to R (c-het. other gene variants)	blade THU7		none specified	inherited haemolytic anaemia	none specified	[79]
1287 (1282)	W premature termination (with c.2330−2_2330−1del)	blade THU7		none specified	GLD	NIFH polyhydramnios bilateral pleural effusions micrognathia bilateral oedema of the calves elevated pleural triglycerides	[95]
1299 (1294)	R to C (with E1344 del)	blade THU7		none specified	NIFH	bilateral pleural effusion generalized skin oedema minor ascites polyhydramnios pregnancy termination anasarca	[96]
1332 (1327)	N to T	beam		none specified	liver biliary pancreas abnormality	none specified	[40]
1344 (1339)	E deletion (with R1299C)	beam		none specified	NIFH	bilateral pleural effusion generalized skin oedema minor ascites polyhydramnios pregnancy termination Anasarca	[96]
1357 (1352)	I to V or M	beam		none specified	DHS	none specified	[46]
1358 (1353)	R to P (or C with A1678V) [96]	beam		increased activity (slower inactivation)	DHS Congenital arm lymphedema	none specified	[46,96,97]
1361 (1356)	Q to R	beam		none specified	anaemia	jaundice, splenomegaly, mild macrocytic, chronic haemolytic anaemia, dessicytes, dyserythropoiesis	[98]
1396 (1390)	S to F	central plug		none specified	angioimmunoblastic T-cell lymphoma	none specified	[99]
1404 (1398)	R to W	lateral plug		decreased opening (mouse PIEZO1)	osteoarthritis	non-syndromic familial osteoarthritis free from acute or traumatic joint injury. erosive hand osteoarthritis and interphalangeal joint osteoarthritis	[54]
1416 (1410)	G to R	latch		none specified	congenital haemolytic anaemia	none specified	[47]
1457 (1451)	A to V	latch-to-clasp		none specified	DHS	none specified	[49]
1458 (1452)	V to A frameshift	latch-to-clasp		none specified	NIFH	none specified	[51]
1496 (1487)	A to V (potential variant)	latch-to-clasp		none specified	NIFH	NIFHa	[66]
1497 (1488)	A to V	latch-to-clasp		none specified	liver biliary pancreas abnormality	none specified	[40]
1519 (1510)	G to P (maybe with Q1910K and R2110W)	clasp		none specified	DHS lymphatic malformation (GLD?)	oedema	[46,52]
1527 (1518)	R to H	clasp		none specified	protective against primary open-angle glaucoma	none specified	[99]
1540 (1531)	S to A (with C513S)	clasp		none specified	NIFH	NIFH Nuchal translucency	listed in [51,53]
1547 (1538)	R to H (with A2227V)	clasp		none specified	NIFH GLD	skin thickening (fetal occiput and nucha) pleural effusions pericardial effusion caesarean delivery at 34 weeks	[51,61]
1566 (1557)	D to G	clasp		none specified	DHS	none specified	[33]
1570 (1561)	V to L (with G1629R)	clasp		none specified	NIFH	none specified	listed in [51,53]
1591 (1582)	Q to P (with E1910K and R2110T)	clasp-to-THU8		none specified	NIFH	none specified	listed in [51,53]
1617 (1608)	T to M (maybe with other gene variants)	clasp-to-THU8		none specified	vasculitis and autoimmune connective tissue disease	colchicine, glucocorticosteroids, azathioprine	[100]
1629 (1620)	G to R (with V1570L)	clasp-to-THU8		none specified	NIFH	none specified	listed in [51,53]
1630 (1621)	E premature termination (hom.)	clasp-to-THU8		decreased expression	lymphedema (GLD?)	head and neck swelling, hydroceles, polyhydramnios, micrognathia, recurrent facial cellulitis, deep vein thrombosis, genital oedema, chylothorax/chylothoraces and bilateral pleural effusions at age 2 years, VVs, bilateral sensorineural deafness, hypothyroidism, mild developmental delay occasional stomatocytes	[70]
1638 (1629)	Y premature termination (with L41del)	clasp-to-THU8		none specified	NIFH	none specified	listed in [51,53]
1671 (1671)	R to W (with R1010H)	blade THU8		none specified	GLD	chylothorax bilateral lower limb oedema	[86]
1678 (1678)	A to V (with R1358C)	blade THU8		none specified	congenital arm lymphedema	none specified	[96]
1684 (1684)	A to V	blade THU8		none specified	brain arteriovenous malformations	none specified	[69]
1712 (1712)	V to M	blade THU8		none specified	adenomatous polyposis	none specified	[67]
1721 (1721)	S to W	blade THU8		decreased protein expression (hom.)	GLD NIFH	pleural effusions fetal demise pregnancy termination	[101]
1732 (1732)	T to M	blade THU8		none specified	DHS	none specified	[33]
1763 (1763)	Y premature termination (with E2392K)	blade THU8		none specified	adenomatous polyposis Er antigen	history of blood transfusion	[67,102]
1764 (1764)	E to Q (with 782 and 808)	blade THU8		none specified	DHS with or without perinatal oedema	cystic hygroma liveborn dysmorphic features redundant nuchal skin, fifth toe clinodactyly, bilateral ventriculomegaly	[35]
1790 (1790)	L to S frameshift (with c.7049+1G>C)	blade THU8		none specified	NIFH	mortality within 1 day of birth polyhydramnios skin oedema pleural effusions. needle drainage of pleural effusion, thoracoamniotic shunt placement intrauterine transfusion	[51,61]
1791 (1791)	M to I	blade THU8		none specified	neuro-developmental disorder	variant predicted to be non-pathogenic	[103]
1797 (1797)	R to C	blade THU8		none specified	DHS	none specified	[50]
1828 (1840)	E to Q	blade THU9		none specified	brain arteriovenous malformations	none specified	[69]
1864 (1881)	R to H (with E2492_ L2493 duplication)	blade THU9		none specified	DHS	none specified	[104]
1877 (1894)	K deletion	blade THU9		decreased activity (faster inactivation, less current)	DHS	none specified	[105]
1898 (1913)	E to D	blade THU9		none specified	DHS	none specified	[50]
1906 (1921)	P to K frameshift (with I2270L)	blade THU9		none specified	NIFH	none specified	[51,59]
1909 (1924)	R to E frameshift (with c.6165−7G>A)	blade THU9		decreased protein expression	NIFH lymphedema	peripheral oedema hydrocele chylothoraces	[106]
1910 (1925)	E to K (with Q1591P and R2110W)	blade THU9		none specified	DHS NIFH	oedema [52]	[46,51]
1925 (1941)	R to W (may be with E2322 premature termination)	blade THU9		none specified	DHS NIFH	skin oedema bilateral hydrothorax ascites intrauterine death at 21 weeks thickened subcutaneous tissue placenta enlarged, thickened, with light and bright membranes, diffuse oedema of chorionic plate and villi atrophic epidermis, oedema, dilated vessels recurrent paternal stomatocytosis (het.)	[46,107]
1943 (1959)	R to Q or W	blade THU9		increased activity (slowed inactivation)	DHS inherited bone marrow failure	none specified	[44,105]
1955 (1971)	R to C	blade THU9		none specified	adenomatous polyposis myelodysplastic syndrome	none specified	[67,94]
1978 (1994)	G to D (with R2335Q)	blade THU9		decreased mechanical sensitivity	lymphedema	NIFH polyhydramnios oedema of limbs (mainly feet), face and scrotum pleural effusions, chylothoraces maldescended testis left side perihepatic ascites no dysmorphic features	[80]
1988 (2004)	A to V	blade THU9		increased activity (slowed inactivation)	malaria resistance	none specified	[71]
1994 (2010)	S to F	blade THU9		none specified	DHS	none specified	[46]
2003 (2019)	A to D or N or T (may be with P2251S [108])	blade THU9		none specified	DHS fetal ascites [108]	in the fetal ascites case: pericardial effusion and pregnancy terminated paternal history of haemolytic disorder and jaundice placenta apparently normal	[46,68,108]
2006 (2022)	V to I or D	blade THU9		none specified	DHS negative association with HbA1c (glycated haemoglobin A1c; >6.5% indicates diabetes)	none specified	[46,109]
2007 (2023)	M to L	blade THU9		none specified	DHS	none specified	[46,110]
2014 (2030)	T to I	blade THU9		none specified	DHS	none specified	[49,110]
2020 (2036)	A to T [97] or V [68] (A to V het [68]).	blade THU9		increased activity (slowed inactivation) A to T	DHS	pseudohyperkalaemia no perinatal oedema	[46,68,97]
2022 (2038)	Y to H	blade THU9		decreased activation	bicuspid aortic valve disease	none specified	[37,38]
2023 (2039)	L to V	blade THU9		none specified	DHS	none specified	[111]
2029 (2045)	G to R (with S1153 premature termination)	blade THU9		decreased surface expression and N-linked glycosylation protein degradation	lymphatic dysplasia (GLD?)	hydrops and bilateral chylothorax swelling of legs and scrotum lymphedema of legs, torso and face pleural effusions intensive care including extracorporeal membrane oxygenation (one sibling)	[38,39,91]
2069 (2085)	V to M	blade THU9		none specified	beta thalassemia	none specified	[112]
2070 (2086)	K to Q (with W1069 premature termination)	blade THU9		none specified	GLD NIFH	stillbirth facial and corporal oedema macroencephaly hypoplastic lungs failure of lymphatic luminal canalization hydroscopic chorionic villi fetal vascular malperfusion	[87,88]
2088 (2104)	R to G	blade THU9		increased activity (reduced threshold for activation) decreased expression (intracellular localization)	DHS	none specified	[105]
2110 (2126)	R to W or Q (with Q1591P and E1910K in NIFH)	anchor		increased activity (R to W)	DHS HbA1c NIFH	oedema [52]	[46,50,52,113,114]
2127(2142)	T to M	anchor		increased activity (slowed inactivation) [97]	DHS HbA1c	pseudohyperkalaemia	[46,68,97]
2151 (2167)	C to R	anchor		none specified	DHS	none specified	[46]
2157 (2172)	K to E frameshift (c-het.)	anchor		none specified	NIFH	NIFH medical termination of pregnancy anteverted nares, dysplastic ears, micrognathia, lymphedema placental hypertrophy and oedema pleural effusion and chylothorax skin oedema, hydramnios, polyhydramnios	[64]
2160 (2176)	P to L	anchor		none specified	DHS	none specified	[46]
2166 (2182)	2166 to 2169 deletion	outer helix		none specified	DHS	none specified	[68]
2169 (2185)	K deletion	outer helix		none specified	DHS	higher mean corpuscular haemoglobin concentration	[115]
2171 (2187)	V to F (with donor splice site variant)	outer helix		decreased expression	lymphedema NIFH	pleural effusions ascites gastro-oesophageal reflux, intestinal lymphangiectasia, granuloma annulare scoliosis oedema resolved rapidly and improved on low-fat diet	[70]
2192 (2208)	L to I	outer helix		none specified	DHS	none specified	[46]
2195 (2211)	S to L (with G253R)	cap (interface with outer helix)		decreased opening (murine PIEZO1 model)	prune belly syndrome	abdominal wall laxity flared ribs dimpled knees dilated bladder bilateral hydroureter surgical urinary bladder drainage and other procedures bilateral hydronephrosis supraventricular tachycardia	[43]
2201 (2217)	V to F	cap		none specified	DHS	none specified	[46]
2225 (2241)	M to R (het.)	cap		increased activity (slowed inactivation)	DHS	none specified	[97,105,116,117]
2227 (2243)	A to V (with R1547H)	cap		none specified	NIFH	none specified	[51]
2228 (2244)	Q premature termination (with E755 premature termination or V1458A frameshift)	cap		decreased expression	lymphedema (GLD?)	NIFH pleural effusions polyhydramnios death *in utero* at 34 weeks amyoplasia of the diaphragm mild generalized oedema with pleural effusions and periorbital oedema chylothorax/chylothoraces cupped simple ears, epicanthic folds, micrognathia, atrial septal defect gastro-oesophageal reflux short stature, pectus excavatum, genital oedema, splenomegaly	[70,118]
2245 (2261)	R or K (hom.)	cap		Yoda1 responsive	Er antigen GLD	NIFH, fetal growth restriction, postnatal demise	[102]
2251 (2267)	P to S (with A2003D)	cap		none specified	fetal ascites	pericardial effusion pregnancy terminated paternal history of haemolytic disorder and jaundice	[108]
2266 (2282)	K to I	cap		none specified	DHS	none specified	[50]
2270 (2286)	I to T (hom. or with E829V [80] or with P1906K frameshift [59]	cap		decreased mechanical sensitivity	GLD	no NIFH late-onset bilateral oedema of lower limbs and scrotum hydrocoeles, pleural effusions, chylothoraces, ascites death due to chylothoraces and pleural disease mild or major pericardial effusion (requiring percutaneous drainage), progressing to cardiac tamponade pericardial thickening breathlessness and reduced exercise tolerance VVs with eczema mild microcephaly, short stature, osteopenia	[59,80]
2277 (2293)	L to M	cap		none specified	DHS HbA1c	increased frequency in South Asians compared with European ancestry	[46,113,119]
2302 (2318)	R to H (with V499I or other β-thalassemia variant) Or P	cap		increased activity (increased mechanical sensitivity, no change of inactivation) decreased expression (intracellular localization)	DHS NIFH GLD	oedema	[51,52,105]
2308 (2324)	Q to E	cap		none specified	DHS	none specified	[46]
2322 (2338)	E premature termination (with R1925W)	cap		none specified	GLD NIFH	skin oedema bilateral hydrothorax ascites intrauterine death at 21 weeks thickened subcutaneous tissue placenta enlarged, thickened, with light and bright membranes, diffuse oedema of chorionic plate and villi atrophic epidermis, oedema, dilated vessels recurrent paternal stomatocytosis (het.)	[107]
2323 (2339)	K to T	cap		none specified	DHS	none specified	[49]
2335 (2351)	R to Q (with G1978D)	cap		decreased mechanical sensitivity	lymphedema (GLD?)	NIFH polyhydramnios oedema of limbs (mainly feet), face and scrotum pleural effusions, chylothoraces maldescended testis left side perihepatic ascites no dysmorphic features	[80]
2336 (2352)	R to W (with V598M)	cap		none specified	NIFH	none specified	Listed in [51,53]
2373 (2389)	Q premature termination	cap		none specified	NIFH	medical termination of pregnancy, hepatomegaly placental hypertrophy and delayed villous maturation ascite, pleural effusion and chylothorax	[64]
2390 (2406)	R to Q	cap		none specified	alcoholic liver disease	none specified	[40]
2392 (2408)	E to K (with G2394S or premature termination Y1763)	cap		Yoda1 responsive	Er antigen	none specified	[102]
2394 (2411)	G to S (hom. or c-het. with A2395V or G2399S)	cap		Yoda1 responsive	DHS Er antigen	history of 1 stillborn infant	[50,90,102]
2395 (2412)	A to V (with Q1114E)	cap		none specified	GLD	NIFH Er antigen [90]	Listed in [51,53]
2399 (2416)	G to S (c-het. with G2394S)	cap		none specified	Er antigen	none specified	[90]
2402 (2428)	E premature termination	cap		none specified	VV disease	none specified	[120]
2407 (2433)	E to Q or K (hom.)	cap		Yoda1 responsive	Er antigen	NIFH and intrauterine death at 26 weeks gestation in 1 of 6 pregnancies	[102]
2430 (2456)	P to L (with donor splice site variant)	cap		none specified	lymphedema (GLD) NIFH	bilateral pleural effusions (severe) generalized oedema, oedema resolved in 1 day, epicanthic folds cellulitis, spherocytes died at age 4 weeks	[70]
2433 (2459)	G to R	cap		none specified	DHS	none specified	[46]
2439 (2465)	G to E (hom. or with Q1125 premature termination)	cap		none specified	lymphedema	venous valve anomaly, venous varicosities	[121]
2456 (2482)	R to H	inner helix		increased activity (slowed inactivation) [97,105]	DHS	mild macrocytic, chronic haemolytic anaemia, dessicytes	[46,50,68,97,98,105,116,117]
2456 (2482)	R to C or H (with L939M and F2458L)	inner helix		none specified	NIFH oedema	pleural effusions polyhydramnios generalized oedema at birth chylothorax/chylothoraces gastro-oesophageal reflux, hypothyroid at birth, occasional stomatocytes, webbed neck, periorbital oedema, prune belly	[70]
2458 (2484)	F to L (may be with L939M and R2456C)	inner helix		increased opening (mouse PIEZO1)	NIFH oedema hip osteoarthritis requiring surgery DHS	pleural effusions Polyhydramnios generalized oedema at birth, chylothorax/chylothoraces, webbed neck, periorbital oedema, prune belly gastro-oesophageal reflux, hypothyroid at birth occasional stomatocytes	[46,54,62,70,122]
2461 (2487)	E to K	inner helix		none specified	DHS	none specified	[84]
2464 (2490)	H to P	inner helix		none specified	DHS	none specified	[46]
2474 (2500)	V to M	C-terminal domain		none specified	DHS. HbA1c?	none specified	[46,113]
2488 (2514)	R to Q or G (with G718S)	C-terminal domain		increased activity (reduced threshold for activation) decreased expression (intracellular localization)	DHS NIFH	NIFH	[46,49,68,105]
2489 (2515)	Q to D	C-terminal domain		none specified	DHS	none specified	[46]
2491 (2517)	R to W	C-terminal domain		none specified	DHS	none specified	[46]
2494 (2520)	E to V	C-terminal domain		none specified	DHS	none specified	[110]
2495 and 2496 (2521 and 2522)	duplication (with V2474M)	C-terminal domain		none specified	DHS	none specified	[46,49,79]
2496 (2522)	E to ELE	C-terminal domain		increased activity (slowed inactivation)	DHS NIFH	perinatal oedema pseudohyperkalaemia hereditary high phosphatidylcholine haemolytic anaemia	[97,105,110,123]
2502 (2528)	K to R	C-terminal domain		decreased activation ecreased opening (mouse PIEZO1)	bicuspid aortic valve disease DHS Osteoarthritis	non-syndromic familial osteoarthritis free from acute or traumatic joint injury. erosive hand osteoarthritis and interphalangeal joint osteoarthritis	[37,50,54,124]
2506 (2536)	L to F	C-terminal domain		none specified	inherited bone marrow failure	none specified	[44]
2510 (2536)	P to L	C-terminal domain		decreased opening (mouse PIEZO1)	DHS osteoarthritis	non-syndromic familial osteoarthritis free from acute or traumatic joint injury. erosive hand osteoarthritis and interphalangeal joint osteoarthritis	[46,54]
2520 (2546)	K to E	C-terminal domain		none specified	DHS	none specified	[50]

### 1.2. PIEZO1 roles

PIEZO1 has roles in contractile, secretory and many other cell types of all major systems that include adipose, cardiovascular, dermal, gastrointestinal, haematological, hepatobiliary, immune, musculoskeletal, neurological, reproductive, respiratory and urinary organs as well as cancers [8]. The roles are sometimes inferred based on the responses of cultured cells to the synthetic chemical agonist of PIEZO1 channels, Yoda1 or an analogue of it [24]. Responses to these agonists may not be physiological but studies of *Piezo1* gene-modified mice also support the idea of diverse functions of PIEZO1 in physiology and disease as modelled in the mouse [14,25–30], all of which are likely to be due to mechanical activation of the channels by endogenous forces. Thus, there might be wide-ranging functional importance of PIEZO1 in human health and disease, but direct testing of such a hypothesis is challenging. We do not currently have PIEZO1 modulators that can be investigated in clinical studies. In particular, we do not have suitable PIEZO1 inhibitors to test the roles of PIEZO1 [24]. However, the *PIEZO1* gene is naturally varied in sequence, and this presents opportunities to explore associations between its variation and phenotypes in familial genetic, genome-wide association and candidate gene association studies. Hundreds of *PIEZO1* variants have so far been linked strongly or tentatively to human traits, diseases and susceptibilities to disease or therapies.

### 1.3. Focus of this article

Here, we review reports of *PIEZO1* variants published in the scientific literature, cataloguing 179 variants in table 1. Additional variants and potential association data may be found in databases such as UK Biobank (www.ukbiobank.ac.uk), ClinVar (www.ncbi.nlm.nih.gov/clinvar), AstraZeneca PheWAS Portal (https://azphewas.com) and FinnGen (www.finngen.fi). The *PIEZO1* gene [125] is on chromosome 16 at locus 16q24.3, so variants are autosomal and can be dominant or recessive. Hence, some associations are evident only when there is homozygosity of the variant but there are also associations with heterozygous variants and compound heterozygous variants (i.e. when two different variants occur in the same person, one inherited from each parent). The variants include exonic variants that may affect details of the PIEZO1 amino acid sequence (missense, deletion, insertion and duplication variants) or the integrity of the PIEZO1 protein (premature termination codons, frameshifts and splice site variants). These variants are the focus of table 1. There are also synonymous and intronic variants, the effects of which may arise via gene regulatory mechanisms.

We begin by addressing the three main disease conditions most associated with *PIEZO1* variants, relating to lymphatics, veins and blood. We then consider additional diseases that may arise because of one or more of the main conditions or independently of them. We discuss physiological traits that may be predisposing factors to some diseases or confer survival advantage. We consider what might be learned about how the channels work as structural machines. We address the relevance to human physiology and the apparent paradox of relatively narrow disease phenotypes despite the widespread expression and functional significance of PIEZO1 that is indicated by laboratory studies. We consider the complexities of the genetic data and what the data might tell us about the systems of the *PIEZO1* gene and the PIEZO1 protein. We discuss the potential for predicting and correcting or compensating for its abnormal expression or function, and thus the opportunities for patient benefit. We make conclusions and discuss potential future directions for research on this topic.

## 2. Lymphatics: generalized lymphatic dysplasia and non-immune fetal hydrops

Generalized lymphatic dysplasia (GLD) is a rare form of primary systemic lymphedema characterized by widespread accumulation of fluid within subcutaneous spaces and body compartments resulting from structural or functional abnormalities of the lymphatic system that affect the whole body [126]. It may present antenatally as non-immune fetal hydrops (NIFH) on ultrasound scan [126]. It is defined as abnormal fluid accumulation in at least two body compartments including pericardial or pleural effusion, ascites and subcutaneous oedema. Immune fetal hydrops is secondary to red blood cell (RBC) allo-immunization whereas the aetiology of NIFH includes congenital infection, cardiovascular and genetic abnormalities. The hydropic changes occur due to an increase in interstitial fluid or impaired lymphatic drainage. Therefore, the term lymphatic-related fetal hydrops may be used to refer to NIFH of lymphatic origin [126].

### 2.1. PIEZO1 disruption identified by familial genetics

**Figure 4.**
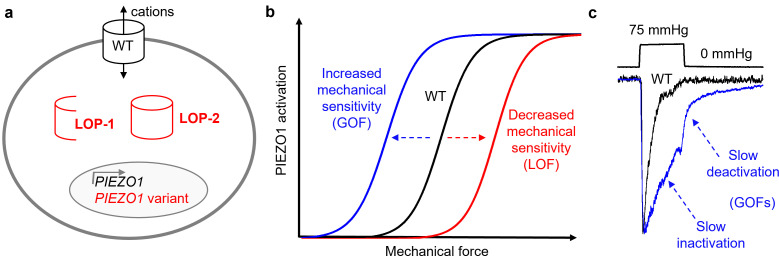
*PIEZO1* variant effects. Effects of variants that cause loss of PIEZO1 expression or function are depicted in red, and those that increase PIEZO1 function are depicted in blue. Non-variant wild-type (WT) is indicated for comparison. (a) Illustrated is the loss or reduction of PIEZO1 expression by fragmentation of PIEZO1 (LOP-1), intracellular trapping of PIEZO1 (LOP-2) or disrupted *PIEZO1* gene transcription (or potentially increased transcript degradation). The WT surface membrane PIEZO1 channel permits flux of cations such as Ca^2+^. (b) Force-response curves illustrating the increasing PIEZO1 channel activation with increasing mechanical force, up to a limit (maximum). Some variants increase the mechanical sensitivity (shifting the curve to the left, which is a type of gain of function (GOF)), while other variants decrease the mechanical sensitivity (shifting the curve to the right, which is a type of loss of function (LOF)). (c) Slow inactivation and slow deactivation as alternative mechanisms of GOF. Outside-out patch data are shown for WT and GOF variant channels overexpressed in HEK 293 cells. The patches were held at a constant voltage of −80 mV. Two recordings are superimposed and normalized to the same maximum current to emphasize the kinetic differences. The pressure steps were to +75 mmHg in both cases.

A distinct recessive form of GLD was identified and referred to as Fotiou GLD [70,126,127] or lymphatic malformation 6 [128]. It is characterized by a high incidence of NIFH and fetal demise. If the fetus survives, there is transient resolution of the oedema, but recurrence of oedema may occur in childhood or later life [70,126,127]. Studies of six families [70] followed by a further three families [80] identified associations with *PIEZO1* variants. These disorders are associated with loss of PIEZO1 protein (LOP) [70,80,91], loss of PIEZO1 channel function (LOF) [80] or both [45,80]. Often, the variants cause premature termination codons early in the reading frame, or frameshifts or deletions resulting in major disruption in at least one allele (table 1), leading to partial or complete loss of PIEZO1 (figure 4a).

Fotiou GLD [126,127] is associated with *PIEZO1* variants that are homozygous or compound heterozygous [70,80,91] (table 1). Severe disease may not be associated with heterozygous single *PIEZO1* variants, so the parents of the GLD suffers may not necessarily suffer from the disease or they may have only mild disease even though they carry a variant [45,70,80]. Therefore, the disease is classed as recessive [70]. There is, however, emerging evidence of dominance in which a single variant heterozygous parent also has the disease [80].

### 2.2. Diversity and potential importance beyond rare disease

*PIEZO1* variant-associated GLD is phenotypically varied (table 1). There may be compartmental effusions of the pleural, abdominal, perihepatic or pericardial spaces. Varying degrees of subcutaneous skin oedema occur, including facial, genital and lower limb regions. Additional features can include chylothorax, webbed neck, prune belly and cranial findings such as craniosynostosis and macrocephaly secondary to ventriculomegaly. Resultant clinical presentations can include respiratory distress, cardiac tamponade, cellulitis and psychiatric morbidity. In pregnancy, there may be placental hypertrophy, chorionic plate oedema and polyhydramnios (table 1). The fetus may die or exhibit increased nuchal translucency, redundant nuchal skin and NIFH. A systematic review of studies identified *PIEZO1* as the most common monogenic cause of NIFH in exome sequencing, with variants observed in up to 10% of cases [51]. Nineteen of 191 cases of NIFH were identified that could be attributed to *PIEZO1* variants.

PIEZO1 is expressed widely by lymphatic endothelial cells, and it is a mechanosensor involved in lymphatic expansion through sprouting lymphangiogenesis and lymphatic valve development [129,130]. Activation of PIEZO1 induces junctional gaps, enhancing lymphatic permeability and drainage, thereby having roles in both physiological lymphatic system formation and function. These mechanisms represent how *PIEZO1* variants leading to either LOP or LOF (figure 4a,b) may underpin lymphatic maldevelopment or malfunction in a fetus affected by NIFH. The findings also suggest widespread importance of PIEZO1 in lymphatics in general, and thus with implications beyond GLD and NIFH.

The varying degrees of penetrance of the variants result in a range of phenotype severities, presenting either antenatally as NIFH or later in adolescence or adulthood. This is further complicated as some variants are associated with both lymphedema and a blood disorder (see table 1 and below). As such, where *PIEZO1* variant GLD is identified antenatally, careful counselling of the phenotypic heterogeneity is required and should be managed by a tertiary fetal medicine unit with genetic counselling.

## 3. Veins: varicose veins

Varicose veins (VVs) are a manifestation of chronic lower limb venous hypertension, leading to the incompetence of venous valves and distorted superficial veins [131]. Venous hypertension, with or without varicosities, due to venous valvular incompetence causes skin changes and predisposes to leg ulcers which adversely affect quality of life, require long-term specialist nursing care and therefore represent a high societal burden. Chronic venous congestion and loss of valvular competence may increase the risk of deep vein thrombosis [131,132]. These problems run in families, suggesting a genetic component in addition to environmental and lifestyle factors [131,132].

### 3.1. Significance identified by genome-wide association

Genome-wide investigation of data for 493 519 people in UK Biobank strongly suggested the association of *PIEZO1* variants with VV disease [133]. Further analysis of UK Biobank data and self-reported VV disease in 408 969 individuals of the 23andMe (www.23andme.com) database also associated *PIEZO1* variants with VVs [41]. Analysis of whole exome sequence data in UK Biobank suggested associations of predicted disruptive *PIEZO1* variants with VV disease [134]. Analysis of data for 131 918 participants in the Geisinger MyCode Community Health Initiative associated VV disease and vein ablation procedures with heterozygous *PIEZO1* premature termination and missense variants [120]. A further study of data in UK Biobank and FinnGen strongly associated *PIEZO1* variants with VVs [135] with stronger association in females [135]. Therefore, the findings suggest that PIEZO1 is important in lower limb venous structure or function and that *PIEZO1* variants, even heterozygous single variants, disrupt the venous structure and its function.

Sequence analysis algorithms have suggested that *PIEZO1* variants associated with VVs are disruptive but there is relatively little direct laboratory evidence for this. One variant generates a premature termination codon in the sequence encoding the cap region (table 1), which suggests that the ion pore region would be damaged, and this is consistent with a disruptive effect.

### 3.2. VVs and lymphedema

There are relationships between VVs and lymphedema. In *PIEZO1* variant-associated GLD, venous varicosities are reported (table 1), and VVs have been associated with another type of lymphedema, called lymphedema distichiasis [131]. Moreover, lymphedema can be caused by chronically increased pressure in the leg, although the mechanisms are not entirely clear. Higher pressure leads to venous dilatation and valve incompetence and varicosities. This could reflect inabilities of the veins to constrict and remodel in response to a higher pressure. Therefore, perhaps not surprisingly, higher venous pressure is associated with peripheral lymphedema, and higher rates of lymphedema have been detected in a VV cohort [120]. This type of lymphedema, secondary to VVs, is important because once established, it can be difficult to resolve clinically, although VV stripping may be helpful [136].

The relationship between VVs and lymphedema is complex, but we suggest two effects. When the PIEZO1 disruption is strong, there may be early onset lymphedema and then late onset VVs that are both direct independent consequences of PIEZO1 disruption. When the PIEZO1 disruption is partial, there may be only late-onset disease that is characterized by VVs and lymphedema; in this case, the lymphedema could be both a direct consequence of the PIEZO1 disruption and the increased venous pressure. When there is partial PIEZO1 disruption and only late-onset disease, environmental and lifestyle factors are likely to be important contributors to the outcome.

## 4. Blood: dehydrated hereditary stomatocytosis

Dehydrated hereditary stomatocytosis (DHS) is an autosomal dominant mild or moderately severe haemolytic anaemia in which the RBC plasma membrane is leaky to cations such as K^+^ and hence the RBCs dehydrate and may adopt a mouth-like, stomatocyte, shape [137,138].

### 4.1. PIEZO1 enhancement identified by familial genetics

Variation in chromosome 16q23-q24 was identified as a potential cause of DHS, leading to a focus on *PIEZO1* and the association of DHS in two families with heterozygous or homozygous *PIEZO1* missense variants that encode M2225R in the PIEZO1 cap and R2456H in the inner helix. Further studies identified other DHS families and associated *PIEZO1* variants [68,97] (table 1). In many cases, the variants are missense, changing a single amino acid in PIEZO1, but there are also amino acid deletions, insertions and duplications (table 1).

Some DHS-associated variants have been recapitulated in *PIEZO1* cDNA clones overexpressed in cell lines for patch-clamp recording in the laboratory. The results of these studies suggest that a consequence of the variation is the slowing of the PIEZO1 channel’s inactivation mechanism [97,105] (figure 4c). This is a gain of function (GOF) effect that results in more channel activity and therefore more non-selective cation fluxes across the cell membrane. Part of this cation flux is K^+^ efflux but there is also Ca^2+^ influx, which triggers the opening of K_Ca_3.1 Ca^2+^-activated K^+^ channels encoded by the *KCNN4* gene, further increasing the K^+^ efflux [138]. This suggests that cases of *PIEZO1* variant-associated DHS arise through increased PIEZO1 channel-mediated ion flux and downstream consequences of this through increased Ca^2+^ entry.

In some laboratory studies, DHS-associated variants were found to have no effect on channel function, or they decreased channel expression or its surface localization (table 1). A few of the suggested effects were not replicated in other studies (table 1). For many variants, data are not available on whether channel expression or activity is affected. For some DHS variants that have been studied, no change in mechanical sensitivity was observed [97,105], suggesting that the changes in amino acid residues at the affected positions do not regulate the mechanical sensitivity of the channels. There are exceptions, however. R2088G, R2302H and R2488Q reduced the threshold for mechanical activation (table 1). R2302H had no effect on inactivation. R2488Q caused intracellular trapping of the PIEZO1 protein, which is a disruptive (LOP) effect. The consequence of GOF may exceed that of trapping (LOP) effects, thus conferring an overall increase in PIEZO1 function. It has been suggested that the severity of DHS may depend on the location of affected amino acids in the PIEZO1 structure such that those in the ion pore region are associated with the most severe disease [139,140].

It is challenging to understand how a PIEZO1 channel that often inactivates in less than a second (figure 2b) could be relevant to RBC physiology, which involves sustained mechanical stress and roles lasting many minutes, hours and days. As indicated, however, the inactivation rate of PIEZO1 channels may change and be regulated. Indeed, patch-clamp studies of physiological RBCs from mice revealed that native PIEZO1 channel activity in RBCs is relatively slow and not characterized by fast inactivation [141]. When the M2225R variant was recapitulated in the native mouse *Piezo1* gene, the RBC PIEZO1 channels of these mice were more active than those of wild-type RBCs, consistent with a GOF mechanism [141], but, in the absence of inactivation, the effect of the variant was to reduce deactivation [141] (figure 2c). PIEZO1 channels interact with their environment [8,18], and, as such, data obtained from PIEZO1 channels in overexpression systems do not necessarily indicate the behaviour of native PIEZO1 channels. There is relatively little information on the properties of native variant channels *in situ* in patient RBCs, but these PIEZO1 channels may exhibit continuous (i.e. non-inactivating) behaviour [68].

### 4.2. Hyperferritinaemia

A clinical feature identified in some severe cases of European *PIEZO1* variant-associated DHS was hyperferritinaemia [46,142]. In this condition, there is excess ferritin (an intracellular iron storage protein) and hepatic iron overload that can have serious adverse implications [142]. These effects may not be due to PIEZO1 in RBCs but rather PIEZO1 in tissue-resident macrophages such as Kupffer cells of the liver that mediate phagocytosis, and thus clearance, of RBCs [25]. Intriguingly, the E756del GOF variant (table 1) is associated with iron overload in African Americans who self-report as healthy, suggesting that there are not necessarily obvious adverse implications of GOF [25]. The implications of hyperferritinaemia are made complex by sex, disease and environmental dependencies and other comorbidities that may include VV disease [132].

### 4.3. Er antigens

Extensive investigation identified *PIEZO1* variants associated with the rare and high-incidence Er antigen blood groups Er^a^, Er^b^, Er3, Er4 and Er5 [90,102]. Among the variants are four missense variants that alter amino acid residues in the cap structure, thus presenting extracellular antigens [102]. Some of the variants overlap in associations with DHS and NIFH (table 1).

### 4.4. Haemoglobin A1c

Haemoglobin A1c (HbA1c), a glycated form of haemoglobin that increases in type 2 diabetes, is commonly used as a biomarker of diabetes. It is associated with some of the *PIEZO1* variants, particularly in people of South Asian origin [113,135]. This effect may occur because of altered RBC turnover, as has been noted for other haemoglobinopathies.

### 4.5. Other implications: adverse and beneficial

Other GOF implications, or potential implications are erythrocytosis, β-thalassemia, myelodysplastic syndrome (table 1) and malarial resistance [71]. Sickle cell trait, which may also affect RBC shape, associated with resistance to *Plasmodium*, the causative agent in malaria, prompting investigation of the relevance of DHS-associated *PIEZO1* variants to malaria [71]. A search was performed using Exome Aggregation Consortium data from several sequencing projects and two of the identified variants were found to increase PIEZO1 activity [71] (table 1). One of these variants (E756del) has a high (9–23%) allele frequency in people of recent African descent compared with <1% for other populations [71]. This suggests positive selection in people most exposed to *Plasmodium* [25]. The idea is supported by results from people in Gabon [72], but not Ghana [73], perhaps differing because of geographical effects [73].

### 4.6. Dehydrated hereditary stomatocytosis and lymphedema

Some variants associate with both GLD and DHS [143]. This situation is complicated by compound heterozygosity and lack of information on the functional consequences of the variants in many cases (table 1). GOF may be a common theme here, but further investigation of this matter would be beneficial to inform understanding.

## 5. Other disease and physiological traits

### 5.1. Gain of function

People with the E756del GOF variant were studied for non-RBC effects in a small-scale population study, and they were found to have normal blood pressure and normal body mass index [75]. The recapitulation of human GOF variants in mice has enabled more detailed studies though. The mice exhibited DHS and stomatocytes. As well as iron overload [25], there was mild cardiac hypertrophy and fibrosis [144] and reduced bile flow [40]. Potentially related is the observation that mice overexpressing wild-type (non-variant) PIEZO1 in cardiac myocytes exhibit dilated cardiomyopathy [29]. Based on studies of the F2458L mutation recapitulated in overexpressed mouse PIEZO1, GOF may also associated with osteoarthritis [54] (table 1). Therefore, the consequences of GOF variants may extend beyond RBCs and potentially confer long-term health risks.

### 5.2. Disruption: expectations from mouse studies

Multiple implications of *PIEZO1* disruption are expected based on the results of gene disruption (inhibition) studies in mice that suggest functions of PIEZO1 across multiple systems including, for example, in bone formation [26,145], immunity [27], skeletal muscle function and repair [30,146,147], blood pressure regulation [148,149], adverse reactions of the heart to pressure overload and ischemia [29,150,151] and memory [152]. The first and most striking phenotype of homozygous *Piezo1* gene disruption in mice is embryonic lethality, occurring at about embryonic day 10, apparently because of failed vascular maturation that is normally driven by blood flow from the newly beating heart [14,153]. To study adult phenotypes in mice, therefore, cell type-restricted and both conditional and cell type-restricted *Piezo1* disruptions have been implemented. The outcomes of such studies lie behind many of the proposals for physiological roles of PIEZO1. We cannot perform comparable genetic experiments in people but we may in the future be able to do something similar if we develop PIEZO1-selective inhibitors [24], but, for now, we can look more closely at the phenotypes of people with disruptive *PIEZO1* variants.

Intrauterine fetal demise has been associated with disruptive *PIEZO1* variants and NIFH (table 1). This does not, however, resemble the embryonic lethality in mice, which is seen as growth restriction and embryo readsorption [14]. Early embryonic lethality may be difficult to detect in people if it occurs as unexplained miscarriage. However, whole exome sequencing of pregnancy tissue may reveal newly identified pathogenic variants in patients with normal chromosomal analysis, as has been demonstrated in a consanguineous couple with recurrent miscarriage, where a *PIEZO1* mutation was detected [154]. What is clear, is that there are some people alive who have, to the best of our knowledge, no PIEZO1 [70]. The severe PIEZO1 deficiency in these people may be compensated in some way but, if it is, it is only partially successful compensation or cell type-specific because these people usually suffer NIFH and GLD. In mouse studies, some PIEZO1-null embryos survive at least a week longer than other null embryos *in utero* [14], so there may also be mechanisms for compensation in mice. If large population studies were performed in mice, a few PIEZO1-null survivors may be found in this species too.

### 5.3. Other vascular associations

There are many clinical features of GLD patients and other *PIEZO1* variant carriers that may arise from lymphatic disruption (table 1), or alternatively, they may arise from other physiological roles of PIEZO1 that are similar or comparable to those seen in mice. Some of these features are vascular-related, such as maternal and fetal vascular malformations, venous valve anomaly, double superior vena cava, agenesis of the ductus venosus, bilateral periorbital and conjunctival vascular changes with small punctate haemorrhages, deep vein thrombosis, cardiomegaly and atrial septal defect (table 1). The familial association of disruptive *PIEZO1* variants with a case of multi-organ prune belly syndrome [43] is reminiscent of some aspects of prune belly seen in GLD (table 1). We know from studies of people who apparently do not have lymphedema, that there are potential associations with other putatively endothelial-related events such as cerebral cavernous and brain arteriovenous malformations as well as bicuspid aortic valve disease (table 1).

### 5.4. Non-vascular associations

Other reported clinical features are bone abnormalities that include osteopenia and osteoarthritis (table 1). There are reports of vasculitis and autoimmune connective tissue disease, metabolic dysfunction-associated fatty liver disease, alcohol-related fatty liver disease, liver biliary pancreas abnormality, obesity in a Han Chinese population, inherited bone marrow failure, angioimmunoblastic T-cell lymphoma, SARS-CoV-2 infection, adenomatous polyposis, antidepressant therapy outcome and protection from glaucoma (table 1). There are reports of hepatic dysfunction and jaundice, a suspected case of posterior urethral valve, elevated pleural triglycerides, obstructive sleep apnoea, primary immune deficiency and Asperger syndrome, and there is quite common gastro-oesophageal reflux (table 1). Some of these associations bear resemblances to phenotypes seen in mouse genetic studies but we cannot be sure of the relationships. Detailed investigation of the associations is often difficult because of the rarities and diversities of *PIEZO1* variants, the polygenics of some of the associated complex diseases and the many potential environmental factors.

### 5.5. A hypothesis for single variant heterozygous disruption

More common than the PIEZO1 knockout or compound heterozygous disruption seen in GLD sufferers, is a single disruptive heterozygous *PIEZO1* variant in which there is partial disruption or deficiency of PIEZO1. In these people, who are apparently healthy, there may effectively be haploinsufficiency in which there is only about half the normal amount of PIEZO1. Examples can be found in table 1, but these may reflect only a tiny fraction of such variants because many more variants are seen in genetic databases without knowledge of the consequences. In a few cases, we know that such variants are disruptive, yet the affected people do not have NIFH or GLD. This is reminiscent of heterozygous PIEZO1 knockout mice that also appear healthy and grow similarly to wild-type mice [14]. There has been relatively little study of the heterozygous mice and only at a young age, but disturbed endothelial cell properties were seen in their reduced alignment to the direction of blood flow and lesser phosphorylation of endothelial nitric oxide synthase [14]. Effects of this type are expected to increase the long-term risks of cardiovascular disease and potentially other diseases such as vascular malfunction-related dementia. Is this also the situation in people? Might haploinsufficiency make people vulnerable to environmental factors such as elevated blood cholesterol, which is a PIEZO1 inhibitor [155]?

### 5.6. Trait associations

Traits are distinguishing qualities or characteristics that typically belong to one person or a group of people. Many of them are not diseases but they may confer advantages or disadvantages, and they may confer an increased or decreased risk of disease depending on the context. *PIEZO1* variants associated with physiological traits in people. Notable among them are the strong associations with standing and sitting heights [135], which are, for example, relevant to conditions such as VV, which is more common in taller people [133,135]. There are associations with other physical factors such as forced expiratory volume [135], tendon stiffness and jumping ability [76–78]. Associations are suggested with body mass index, obesity and dyslipidaemia [40,113,156], which are consistent with observations in *Piezo1* genetically modified mice [28,40] and relevant to lymphedema and VVs [131,132,157].

## 6. Potential origins of restricted phenotypes

### 6.1. A model

**Figure 5.**
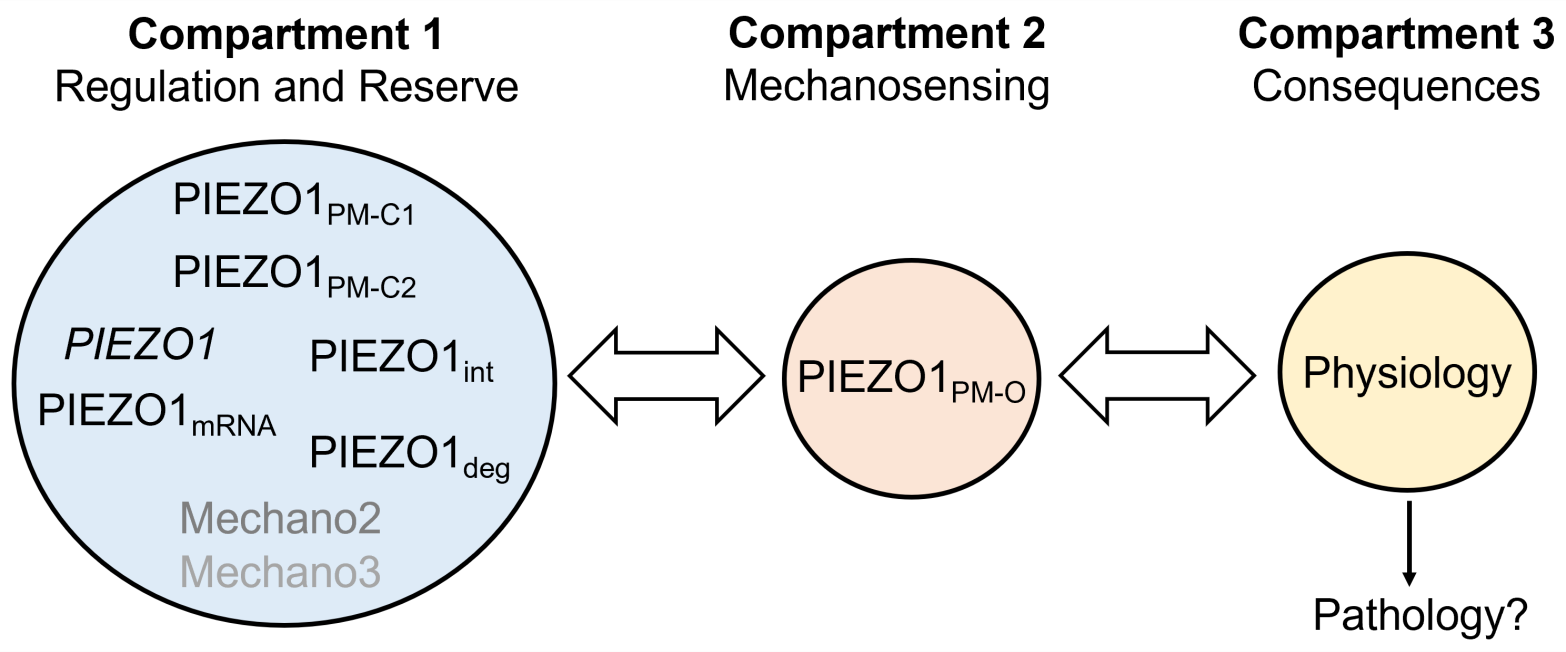
Diverse phenotype model. In the model, there is compartment 1 for regulatory and reserve systems that try to ensure compartment 2 is supplied with suitable mechanical sensors for physiological force sensing. Compartment 1 contains: *PIEZO1* (gene) regulation, which may occur, for example, via microRNAs and transcriptional enhancers and silencers; mRNA encoding PIEZO1, the abundance of which may be modulated and which is available for translation and thus production of new PIEZO1 (protein); PIEZO1 in intracellular membranes (PIEZO1_int_), which may translocate to the plasma membrane (PM); PIEZO1 at the PM in closed states C1 or C2 due to mechanical insensitivity or inactivation, or be degraded (PIEZO1_deg_); and other mechanical force sensors (Mechano2 and Mechano3), an example of which may be PIEZO2. Compartment 2 comprises PIEZO1 at the PM that is available for opening (O) in response to mechanical stimuli (PIEZO1_PM-O_). Consequences are divided into physiology and pathology effects occurring via compartment 3. Physiology effects, occurring via the normal biochemical signalling pathways of cells and tissues, do not necessarily increase if PIEZO1 increases. Pathology, possibly involving non-physiological pathways, may be triggered by strong depletion or mild excess of PIEZO1, depending on the cell type.

Why are the most prominent phenotypes those of lymphatics, lower limb veins and RBCs? Why are there cases of GOF variants with apparently no lymphatic effect, and cases of disruptive variants with apparently no RBC effect? Here, we outline a model of how such consequences might arise (figure 5). In compartment 1 of the model, there is a system for mechanosensor production, regulation and degradation with a store of readily available but not necessarily used mechanosensors. This compartment serves compartment 2, the active site of mechanosensing, which is a component of compartment 3, which is where physiological effects arise. Compartment 1 contains *PIEZO1* (gene) and PIEZO1 (protein) in different stages of readiness as well as alternative mechanosensors Mechano2 and Mechano3 that might comprise PIEZO2, for example. These elements exchange between compartments 1 and 2 according to need.

### 6.2. Analogy

Compartment 1 can be compared with a factory and a warehouse for anemometers, which detect wind speed. It has systems for the exchange of products with compartment 2, which is an element of compartment 3, a power station that measures wind speed in a wind turbine system. In this analogy, compartment 1 contains anemometer type 1 in various stages of production and assembly as well as other devices used to measure wind speed, should anemometer type 1 not materialize. There is communication between the compartments that maximises efficiency and tries to ensure suitable outcomes such as the production of the required amount of wind-driven electrical power. Inadvertent alteration in an anemometer’s sensitivity to airflow velocity through a manufacturing error may be detected if there is a system in place to detect it (e.g. the power station may have four anemometers that are expected to give the same readings within a defined tolerance, so one reading out of range may lead to replacement of the presumed faulty anemometer).

### 6.3. Gain of function in the model

With *PIEZO1* GOF variants, some of the mechanosensors in compartment 2 may overreact to the mechanical forces experienced by the cells. Compartment 1 may be able to compensate for this problem by producing less PIEZO1. If a mechanism for producing less PIEZO1 is transcriptional, cell types without nuclei (e.g. RBCs) will be less able to compensate, and so a phenotype will arise that is dominated by the effect on RBCs, and the most obvious phenotype will be anaemia.

### 6.4. Loss of expression or function in the model

With homozygous or compound heterozygous disruptive (LOP and LOF) variants, PIEZO1s may not be able to provide mechanosensing. Compartment 1 may try to compensate by delivering an alternative mechanosensor (e.g. PIEZO2). In some cell types (e.g. lymphatic endothelial cells), the alternative may not be sensitive enough to detect the required mechanical forces (e.g. the low flow and low pressure of lymph), in which case, the most obvious phenotype will be lymphedema. In other cell types, the substitute mechanosensors may be sufficient, or insufficient only in certain conditions (e.g. when there is a cofactor or comorbidity).

### 6.5. Partner proteins

Another control on the contribution of PIEZO1 is its assembly with other proteins. These proteins include but are not limited to the cell adhesion molecules CDH1, CDH5, PECAM1 and CADM1 [20,158,159], and the MyoD family inhibitor domain-containing protein MDFIC [19]. The expression and importance of these proteins vary in different cell types, and so we can speculate that *PIEZO1* variants may affect the interactions of some of these proteins with PIEZO1, thereby creating cell type-specific effects. Variants in the genes that encode the interacting proteins may likewise affect PIEZO1 expression or activity. A *MDFIC* variant has been associated with lymphedema [160]. *PECAM1* variants have been associated with lymphedema [161] and *CADM1* variants have been associated with endothelial dysfunction and venous thrombosis [162,163]. It is unknown if these associations are related to interactions with PIEZO1.

## 7. Understanding the effects of variants on PIEZO1 channel properties and *PIEZO1* expression

Variants that, for example, insert a premature termination codon and thus truncate the PIEZO1 protein are relatively easy to understand but others, such as missense variants that affect the amino acid sequence of the channel and thus potentially the channel properties are more difficult to understand and are often hard to interpret clinically. Are the channel properties affected? Is channel activity increased or decreased?

### 7.1. Patch-clamp electrophysiology and intracellular Ca^2+^ assays

Ideally, we would measure PIEZO1 channel activity non-invasively in specific systems of people such as the lymphatics, but this is not currently possible. Alternatively, we can isolate cells from a patient’s blood or tissue sample and then measure PIEZO1 activity in these cells by patch-clamp, applying a mechanical stimulus to activate the channels. This may be possible but is often challenging and laborious. Even for the relatively accessible RBC, there are few data of this type reported [68]. The development of automated planar patch-clamp methods that incorporate a mechanical stimulus, including for RBCs, has the potential to overcome some of these limitations and transform the evaluation of patient cells [102,164–166]. Intracellular Ca^2+^ assays can alternatively be used to detect Ca^2+^ influx through PIEZO1 channels, but even when mechanical stimuli are implemented in such assays, the information quality for the channel properties is inferior to that from patch-clamp assays.

To avoid requiring a patient sample, it is possible to recreate the variant in a tractable cell line by gene editing, thereby generating a model system that can be studied by patch-clamp. Substantial investment of funds and researcher time is, however, required for every variant. Even if the cell line is successfully made and validated, the subsequent patch-clamp recordings from the native channels can be difficult because of the small and variable nature of the PIEZO1 channel signals and the presence of other ion channels in the same cells that may complicate the data interpretation.

At the present time, the almost exclusively used method for determining the effects of variants on channel properties is the generation of the variant artificially in a cDNA clone by site-directed mutagenesis, followed by its overexpression in a host cell line for subsequent patch-clamp recording. This method is also challenging, and the ionic currents observed can be highly variable. It is quite common to use the mouse PIEZO1 channel as a surrogate of the human channel because it can be more suitable for robust recordings, and it is better understood and more widely studied. Despite the challenges and limitations, this method generates useful data for important channel parameters such as the mechanical sensitivity.

### 7.2. Amino acid variations that affect channel activity

The overexpression approach described above, whether using the human channel or a mouse surrogate, lies behind most available data for the effects of variants on PIEZO1 channel function. As detailed in table 1, we know from these data of variants altering residues in THU2 and THU3 that decrease channel activity (S217L, G253R, L322P and R531C), a variant in THU4 that increases channel activity (V598M), variants in THU5 that increase (756Edel or G782S) or decrease (E829V or L939M) channel activity or expression, a variant in the beam that increases channel activity (R1358P), a variant in the lateral plug that decreases channel activity (R1404W), seven variants in the THU9 that increase (R1943Q, A1988V, A2020T, R2088G) or decrease (1877Kdel, G1978D, Y2022H) channel activity, variants in the anchor that increase channel activity (R2110W and T2127M), a variant in the outer helix that decreases channel activity (S2195L), variants in the cap that increase (M2225R and R2302H) or decrease (I2270T and R2335Q) channel activity, variants in the inner helix that increase channel activity (R2456H, F2458L and R2488Q) and variants in the CTD that increase (2496E-ELE) or decrease (K2502R and P2510L) channel activity. Mapping of these variations to a model of the channel suggests vulnerability of the central channel region including the cap, but not a restriction to this region (figure 3). There is no obvious pattern to the sites of these LOF and GOF variants, and there are opposing effects seen from variants in proximity.

The origins of *PIEZO1* missense variant-associated disease are therefore complex and mostly poorly understood at the molecular level. Unless we have the patch-clamp data, we are currently unable to predict whether missense variants would increase or decrease channel activity, or indeed simply have no effect, as has been found for some variants (table 1). We are left, therefore, with needing to experimentally test the effect of each new variant unless we can devise new methods that might more quickly determine the effects or accurately predict them. We are also uncertain whether the overexpression data truly reflects what happens in the human body.

### 7.3. Protein structural biology

Structural studies of the channel might provide a way forward because a protein’s structure determines its function. Structural understanding of PIEZO1 channel function is still in its infancy, but there is progress, and it has the potential for determining effects of variants. High-resolution structural data have been obtained for the E756del and A1988V GOF channels in complex with MDFIC and, albeit slightly less well resolved, for the R2456H GOF channel [140]. While the E756del and A1988V channels had a similar dome to the non-variant wild-type channel, the R2456H channel was more curved, with more contracted blades, although the inner helix was twisted, resulting in a more dilated pore [140]. Lipid density was seen in the pore region of the wild-type channel [140], as had been seen previously in structural data and molecular dynamics simulations for the mouse PIEZO1 channel [23,167]. The lipids have been suggested to seal the pore, inhibiting or preventing the flux of ions [23,140,167]. The E756del and A1988V channel pores also contained lipid density, but the acyl chains were reoriented, which could explain the GOF [140]. There was lipid head group close to R2456, and so this may be critical for the lipid interaction in the pore [140]. These structural data suggest that GOF variants can indeed act by altering the structural configuration of the channel.

### 7.4. Synonymous and intronic variants

At present, we can only speculate about why synonymous and intronic variants are associated with human traits and disease. The most obvious hypothesis is that they act by altering the expression of the *PIEZO1* gene. We have little or no evidence, however, and little or no information about the mechanisms that normally regulate the expression of *PIEZO1*, or the murine gene, *Piezo1*. The associations of these variants can give us ideas about regions of the gene that are important in its regulation by transcriptional enhancers and repressors, and non-coding RNAs such as microRNAs.

## 8. Potential for patient benefit

**Figure 6.**
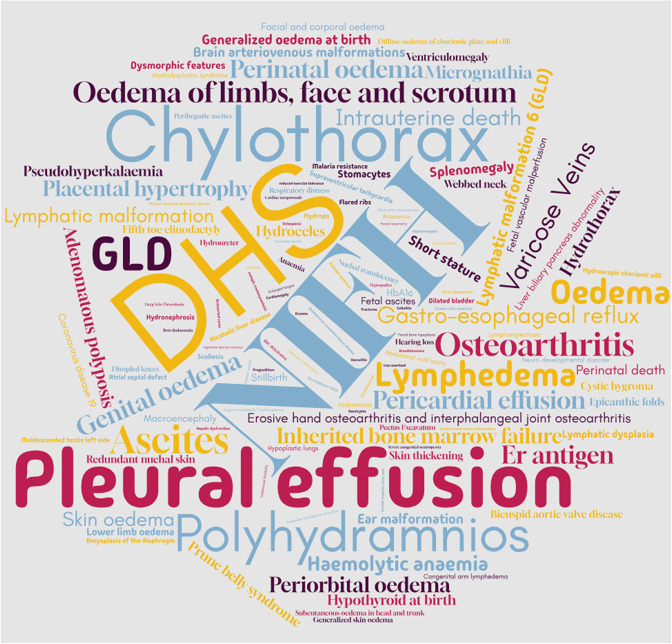
Reported frequencies of *PIEZO1* variant-associated phenotypes: a word cloud for *PIEZO1* variant-associated disease and clinical terms from the ‘primary disease’ and ‘antenatal and other clinical observations, interventions and outcomes’ columns of table 1. The size of a word was determined by the frequency of its occurrence in the table. The colours and fonts of the word groups are random. Some of the phenotypes were manually harmonized by grouping synonymous terms to ensure consistent terminology and accurate weighting (e.g. ‘varicose veins’ and ‘veins varicosities’ were both counted as ‘varicose veins’).

The genetic variant data discussed here, suggest important roles of PIEZO1 in human disease, and so we should consider the possibilities for patient benefits arising from this new knowledge. GLD, NIFH and DHS are prominent in the reports published so far (figure 6). Even in such rare diseases, understanding and manipulating PIEZO1 could generate considerable benefits for conditions that currently have limited treatment options, but, it is our hypothesis, that the benefits of targeting PIEZO1 will extend beyond these conditions and may include conditions that involve PIEZO1 but are not necessarily caused or exacerbated by PIEZO1 variation such as secondary lymphedema arising from factors such as obesity, inflammation, fibrosis and physical injury [157]. Secondary lymphedema is a major problem that affects 250 000 people in the UK [168] and about 230 million people worldwide (World Health Organization data). Improving lymphatic function by stimulating PIEZO1 could therefore have widespread implications. Data from human and mouse studies strongly support the conclusion that PIEZO1 is important in lymphatic function and that beneficial lymphatic effects can be achieved with a PIEZO1 agonist [24,70,129,130,157,169–171].

There may be opportunities for resolving other seemingly unrelated clinical problems, among which we suggest may be traumatic brain injury. Head injury causes significant clinical burden and is the most common cause of injury and disability in people aged 1−40 years in the UK. Each year in England and Wales, approximately 1.4 million people attend emergency departments with head injuries. About 40 000 of these patients show evidence of traumatic brain injury. Many suffer long-term disability and around 0.2% die of their injury. Long-term complications can range from neurological deficits and seizures to endocrine conditions, and mood and behavioural disorders (NICE). Meningeal lymphatic dysfunction exacerbates traumatic brain injury pathogenesis [172], and it has been proposed that PIEZO1 agonists, acting to improve the function of meningeal lymphatics, could reduce excess cerebrospinal fluid and ventricular enlargement [169].

Other conditions such as VVs are even more prevalent. While less high profile, there is importance at a societal level due to chronic disability, adverse quality of life and healthcare costs. Preclinical investigation has supported the idea of targeting PIEZO1 in the treatment of VVs [173]. Since the affected veins are often superficial, topical application of a PIEZO1 modulator might be sufficient, and limit potential safety concerns of a systemic application.

There are also potential clinical implications for the RBC-related variants, for example, in anaemia and malarial resistance, and for the use of Er antigen identification in reducing acute transfusion reactions.

### 8.1. Progress with pharmacology

A review of PIEZO1 pharmacology can be found elsewhere [24] but we note here the encouraging signs that PIEZO1-selective pharmacology is possible, particularly in the case of PIEZO1 agonists [24,45,80]. Other modulators to consider are dietary lipids such as docosahexaenoic acid, which may slow PIEZO1 inactivation and therefore have a GOF, agonist-like, effect [174]; although it may also have a separate inhibitory effect [175]. Mechanical stimulation of the channels by ultrasound [176] might also be considered. Some microRNAs may create agonist-like effects by stimulating *PIEZO1* expression [177]. PIEZO1 pharmacology and other forms of modulation are, however, in their infancy, and only experimental at this stage. The approach is currently unproven with unknown safety implications in the clinical setting.

### 8.2. Progress with computer predictions

As discussed earlier, laboratory methods for determining the effects of variants on PIEZO1 channel expression and function are laborious and potentially financially expensive, contributing to limited progress on the effects of the many variants (table 1). Computer algorithms can generate quick interpretations, but they currently depend on predictions from the DNA sequence, which may have relatively little value in clinical diagnosis when predicting variants of uncertain significance (VUS). While proteome-wide missense prediction algorithms have been developed, they infer only the presence of likely pathogenicity [178]. A potentially more informative computational approach is the development and validation of molecular dynamics simulations, as are emerging for the human PIEZO1 channel in relevant membranes such as those of endothelium and RBCs [155,167]. These simulations are based on lipidomic data for membranes and static structural data from laboratory protein structural biology studies of the channel, the capabilities for which are rapidly improving. There is the prospect that combining such data with molecular dynamics simulation approaches could ultimately enable the prediction of the effects of variants on channel properties and thereby transform opportunities for clinical diagnosis.

## 9. Conclusions and discussion

**Figure 7.**
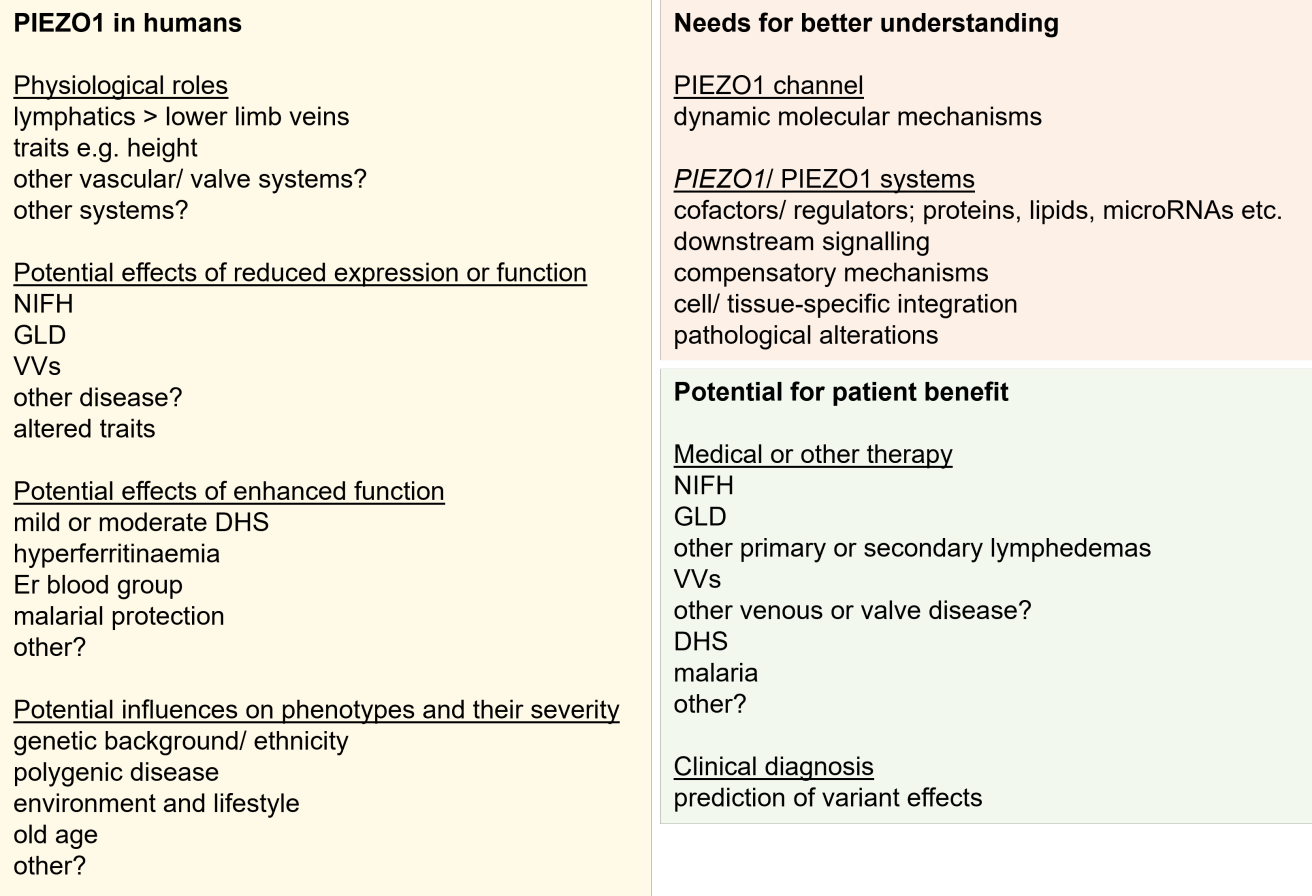
Primary conclusions. Left (pale yellow colour): roles of PIEZO1 inferred from *PIEZO1* variant associations. Top right (pale peach colour): suggestions for future research to address aspects of this biology for which we have particularly limited understanding. Bottom right (pale green colour): promising areas of research for delivering patient benefits based on targeting and further understanding of PIEZO1.

Figure 7 summarizes key conclusions from this review. One conclusion is that the strongest physiological role of PIEZO1 in humans seems to be in the lymphatics, which is based on the observation that lymphedema is the most striking phenotype associated with disruptive *PIEZO1* variants and potentially the most major or even singular disease characteristic in some people who carry such variants. There are almost certainly other roles of PIEZO1, but lymphatic function, with its implications for all major organs, may have overlooked influence on multiple aspects of physiology. Another major physiological role that emerged from these studies is a role of PIEZO1 in the structural resilience of superficial leg veins. This role may have little or no impact in early life, but it is of considerable interest for societies with large ageing multi-morbid populations. The roles of PIEZO1 in lymphatics and leg veins probably both reflect its significance in endothelial cells [14,135] and in turn the roles these cells have in vascular permeability and the control of unidirectional fluid flow mediated by valves.

Unlike the essential lymphatic and venous roles, PIEZO1 seems to be mostly non-essential in RBC physiology. Anaemia is often not a feature of *PIEZO1* variant-associated NIFH or GLD in which we know PIEZO1 is disrupted. RBCs may not depend as much as lymphatics on PIEZO1 because of their multiple diverse mechanisms for membrane cation permeability [179]. The excess cation permeability inadvertently conferred by *PIEZO1* GOF variants nevertheless may disturb RBC physiology. The significance of the disturbance for health varies substantially from the relatively benign and even beneficial, to the quite seriously adverse. The variability may be explained by varying genetic backgrounds that may in some instances make people vulnerable to *PIEZO1* GOF, such as variations in other genes, for example, *KCNN4*.

Despite the broad expression of PIEZO1 and the multiple phenotypes of PIEZO1-disrupted mice, PIEZO1 also seems to be non-essential in other human physiology. But this does not mean that PIEZO1 variation is without other consequences, or that PIEZO1 modulators (e.g. PIEZO1 agonists) would be without adverse effects if administered to people. As we discuss in this article, there are already many potential additional associations of *PIEZO1* variation. We expect that as studies on this topic continue, additional importance in humans will be recognized. In future studies, we suggest the importance of focussing studies on age-onset cardiovascular and musculoskeletal diseases. Tissue stiffness and other mechanical strains increase with age, and we know that PIEZO1’s role in iron homeostasis is age-related [25]. Iron overload could have broad implications for diseases that are more common in older people such as VVs [132], and arthritis, liver damage and heart failure [25]. We should also consider the importance of lipid homeostasis in *PIEZO1* variant-associated phenotype because of the impacts of lipids on PIEZO1 channel function [18,174] and the roles of PIEZO1 in whole body lipid homeostasis [40]. We should also consider the health implications of PIEZO1 haploinsufficiency caused by disruption to one of the *PIEZO1* alleles because of the vulnerability it may confer to disease through a reduced reserve of PIEZO1 channels.

In summary, we conclude that PIEZO1 is important in human health and disease, and that studies of the effects of *PIEZO1* variants have clinical relevance that is only just starting to be broadly realized and which can in parallel advance understanding of how mechanical force sensing works in humans and eukaryotic biology as a whole.

## Data Availability

This article has no additional data.
